# Pet Owners’ Attitudes and Opinions towards Cat and Dog Care Practices in Aotearoa New Zealand

**DOI:** 10.3390/vetsci10100606

**Published:** 2023-10-04

**Authors:** Rachel Forrest, Maria Pearson, Leena Awawdeh

**Affiliations:** 1 Te Pūkenga—Eastern Institute of Technology, Hawke’s Bay, 501 Gloucester Street, Taradale, Napier 4112, New Zealand; 2 Independent Researcher, Hawke’s Bay 4294, New Zealand; 3 Western Sydney University, Richmond, NSW 2753, Australia; l.awawdeh@westernsydney.edu.au

**Keywords:** attitudes, Aotearoa, cat, companion animal, dog, New Zealand, opinions, pet owners, practice, shock-collar, survey, veterinary

## Abstract

**Simple Summary:**

This research aimed to explore cat and dog owners’ attitudes towards various pet care practices in Aotearoa New Zealand (NZ). An online survey was conducted from January to March 2019, and 2358 people responded to the pet care questions. The study revealed that almost all respondents felt that providing adequate housing, regular worming and flea treatments, microchipping, and vaccinations were important pet care practices. Fewer respondents felt that regular veterinary clinic visits were important, and some did not appear to understand that breeding for certain looks causes health problems for animals. Characteristics of pet owners, such as gender, ethnicity, age range, having children, having a rural upbringing, and place of residence, were associated with particular attitudes towards the various practices. Knowing about these factors will help develop strategies that ensure everyone understands what pet care practices are important for responsible pet ownership. Our findings emphasize some of the complexities that underlie NZ pet owners’ attitudes and opinions towards various pet care practices. The findings underscore the need for additional research, culturally appropriate and effective educational resources, and customized strategies to encourage responsible pet care to ensure animals, along with their owners, live good lives.

**Abstract:**

This research aimed to explore cat and dog owners’ attitudes and opinions towards various pet care practices in Aotearoa New Zealand (NZ), and determine what factors were associated with them. An online survey composed of both closed and open-ended questions was administered from January to March 2019. A total of 2358 respondents answered the questions on pet care practices. Of these, 37.5 percent (*n* = 885) were both dog and cat owners, 28.0 percent (*n* = 652) were cat owners, and 26.0 percent (*n* = 609) were dog owners, while 9% (*n* = 212) of respondents did not own a cat or dog at the time of taking the survey. The study revealed that most respondents (>90%) acknowledge the importance of providing adequate housing, regular worming and flea treatments, microchipping, and vaccinations for their pets. Notably, demographic factors such as gender, ethnicity, age range, having children, having a rural upbringing, and place of residence influenced owners’ attitudes, with those towards cat care practices varying more than those for dogs. The study highlights the need for responsible pet ownership interventions considering these demographic factors. The study identifies a knowledge gap among pet owners concerning the importance of regular veterinary visits and the impact of breeding for certain looks on animal welfare. The use of shock collar devices provoked varying opinions on their usage for training and behavior modification. The study suggests that enhancing pet owners’ knowledge is pivotal for responsible pet ownership. Overall, the findings emphasize the need for tailored interventions that account for demographic variations for promoting responsible pet ownership and animal welfare. The findings underscore the importance of improving access to veterinary health care teams, especially in rural areas, and of providing culturally appropriate education resources for both pet owners and veterinary health care teams.

## 1. Introduction

Globally, companion animals have become an integral part of modern society. They offer emotional support and companionship and also work as service animals [1,2]. The prevalence of companion animals varies across different countries due to several factors, including cultural, economic, and social factors. The highest prevalence of companion animals was reported in the United States, with 70% of households having a pet [3,4], followed by 64% of Aotearoa New Zealand (NZ) households [5]. NZ has 4.6 million companion animals, including cats, dogs, birds, rabbits, and other animals. Among these, dogs and cats are the most popular pets, with an estimated population of 525,000 dogs and 764,000 cats, respectively [5]. Cats are, therefore, the most popular companion animal in NZ, with 41% of households sharing their homes with cats [6,7]. Unlike other developed countries, the prevalence of companion animals in NZ is higher in rural areas than in urban areas, with 52% of rural households owning cats compared to 36% in urban areas [5]. It is estimated 90% of owned cats in NZ roam freely without being monitored by their owners, which can contribute to predation and risk behaviors [8], resulting in negative impacts on society.

Companion animals exert both positive and negative impacts on society. On the positive side, companion animals provide emotional support and companionship, improving both mental and general health [9]. They are also used as service animals, such as guide dogs for the blind and hearing dogs for people who are hearing impaired [5]. In addition, companion animals help children to develop empathy, responsibility, and social skills [5]. On the negative side, there are issues such as pet overpopulation, animal cruelty, and neglect [9]. In addition, pet ownership can potentially increase the spread of zoonotic diseases [10]. Proper care and hygiene practices can reduce the risk of these diseases, but they remain a significant human and animal health concern [11]. Pet owners need to be aware of the potential risks of owning pets and act appropriately to protect themselves and their families [12,13,14]. Companion animals also have a significant economic impact on society since the pet industry is a multi-billion-dollar industry that includes pet food, pet care products, and veterinary services [3,5]. In NZ, the pet industry generates over $1 billion in revenue annually and provides employment opportunities for over 2500 people in the pet care industry [5]. 

The continued prevalence of companion animals in NZ society highlights the need for proper pet owner education to mitigate the negative impacts associated with cat and dog ownership [9]. Along with other organizations, veterinary health care teams (including, but not limited to, veterinarians, veterinary nurses, and veterinarian technicians) can play an essential role in addressing these issues by promoting education regarding husbandry, animal behavior, responsible pet ownership, and the effects of pets on the environment [9]. These organizations need to emphasize the significance of keeping companion animals safe, healthy, and happy and of reporting any animal abuse or neglect to law enforcement officials and social service agencies. However, to provide appropriate education, it is necessary to understand what factors are associated with attitudes towards various pet care practices. Even though 64% of NZ households have a pet [5], there is a dearth of studies exploring the attitudes and opinions of pet owners in NZ towards pet care practices. Given that NZ hosts a diversity of cultures, fosters a strong conservation and biodiversity focus, and has farming as a primary industry, there are likely to be differing values placed on pet animals, such as dogs and cats, which will be reflected in pet owners’ attitudes and opinions. Such data is necessary to inform educational interventions aimed at improving pet ownership attitudes, knowledge, and practices to improve animal welfare and human well-being. Therefore, the current study used the relevant data collected from the Furry Whānau Wellbeing project, funded by the NZ Companion Animal Trust (NZCAT) to explore pet owners’ attitudes and opinions towards cat and dog care practices in NZ and answer the question, “What are the factors that influence these attitudes and opinions?” Knowing about these factors will help develop strategies that ensure NZ pet owners understand what pet care practices are essential for responsible pet ownership.

## 2. Materials and Methods

The data were gathered as part of the 2019 NZ Pet Survey completed by adults living in NZ as described in Forrest et al. [15], which was administered between 8 January 2019 and 31 March 2019. The survey collected demographic data (Supplementary Table S1) such as gender, ethnicity, age group, local government region and type of residence (urban, rural, etc.), type of childhood residence (e.g., urban, rural), household income range, highest level of education, and household composition (number of children and adults). Forrest et al. [15] detail the survey’s design, including all of the questions, along with the method of survey distribution. Below are the questions specific to pet owners’ attitudes toward the health and care of cats and dogs:

- Please choose the option (Strongly agree, Agree, Neutral, Disagree, Strongly disagree) that most closely describes how you feel about the following statements. Dogs should: have regular vet checks; have up-to-date vaccinations; have up-to-date flea treatments; have up-to-date worming treatment; be desexed; be micro-chipped; be bred for particular looks; not have their appearance modified (e.g., tail docking, ear cropping, branding, tattooing); have a specialized diet from a pet shop or vet clinic; not be trained using behavior modifying collars (e.g., shock, spray, check chain); have ribs, hips, and a spine that are not visible but are easily felt; have access to adequate housing. Please provide further explanation if required.
- Please choose the option (Strongly agree, Agree, Neutral, Disagree, Strongly disagree) that most closely describes how you feel about the following statements. Cats should: have regular vet checks; have up-to-date vaccinations; have up-to-date flea treatment; have up-to-date worming treatment; be desexed; be micro-chipped; be bred for certain looks; have their appearance modified (e.g., branding, tattooing); have a specialized diet from a pet shop or vet clinic; have ribs, hips, and a spine that are not visible but are easily felt; have access to adequate housing. Please provide further explanation if required.

The quantitative data were analyzed using descriptive statistics (percentages) to show the respondents’ option choices and for each of the statements, cross-tabulations along with Chi-square and z-tests (α = 0.05) were used to explore if the respondent’s answer choices were associated with gender, ethnicity, age range, household income bracket, qualification level, whether or not they had a child or children, whether or not they had a rural upbringing, or whether they were currently living in a town/city. The IBM SPSS Statistics (version 25) software was used to conduct all the statistical analyses. An inductive approach was used to identify emergent themes from the qualitative data [16] by two of the researchers, independently. The themes were then compared and consolidated.

This study explored the attitudes listed in the questions above with the exception of those about appropriate body condition and specialized pet food, the results of which have been presented and discussed in previous publications [6,17].

## 3. Results

### 3.1. Demographic Description of the Respondents

Our previous papers [6,15,17,18] provide a full demographic profile of all respondents (*n* = 2744), along with a breakdown by cat and dog ownership. Among the survey respondents, there was an under-representation of men (7.7% versus 49.4% in NZ 2018) and Māori (8.3% versus 16.5%). Due to this disparity, local government regions could not be included in any of the statistical analyses as some did not have Māori and/or male representatives.

### 3.2. Attitudes towards Dog Care

All survey respondents (including non-dog owners) were asked to respond with their level of agreement about statements regarding dog care (Table 1, Supplementary Figure S1). For each statement, there were between 2280 and 2293 responses. Table 1 shows that nearly all the respondents (>90%) either agreed or strongly agreed that dogs should have access to adequate housing, have up-to-date worming, be microchipped, and have up-to-date vaccinations and flea treatments. More than three-quarters of the respondents (>75%) also agreed or strongly agreed with regular veterinarian checks, desexing, no appearance modification, and correct body condition. Almost 80% of respondents disagreed or strongly disagreed with dogs being bred for a certain look.

The following comment summarized the collective sentiments of many with regard to veterinarian checks and flea and worm treatments:

*They should have veterinarian checks and flea and worm treatments etc., when needed. But if you have experience in what to look for health-wise,* i.e., *keeping an eye on their weight, energy, behavior changes, water consumption, physical changes* etc. *and don’t want to use chemicals on them all the time (flea worm treatments etc.). I don’t think it should be a constant thing to do. As long as your checking and looking out for them and get them treatment/checked with any concerns or if it’s been a long period since they were checked or they are elderly etc.*

Several of the respondents’ comments highlighted the perception that worming and flea treatment may not always be necessary; for example, “In Southland, fleas are not a problem, so we don’t use a flea treatment. We would if our dog/s needed one though,” and, “Worming should be given on the advice of a veterinarian to avoid resistance.” Vaccinating young dogs was viewed as important, while some thought adult dogs were over-vaccinated; for example, “Puppy vaccination are super important but we over vax adult dog/s.” One respondent commented, “Veterinarian care where I live is all owned by one company. It’s very expensive and they often pressure people into unnecessary vaccinations and products.” Concerning microchipping, several respondents thought that a tattoo of the number inside the ear during desexing or while an animal was under anesthesia would be beneficial if the microchip fails. The latter was evidenced by this comment: “As an owner of hunting dogs that are a target for thieves, we have tattooed and microchipped. Our microchips have failed in the past, so we require a fallback identification.”

Another common theme in the comments was that all “breeders should be registered, and all other dogs should be desexed to prevent the number of strays and puppy farms around.” A common perception regarding desexing was that it was being done when the animal was too young; for example, one respondent wrote, “Desexing is done far too early; again, the evidence shows you should wait for the dog to fully mature, so their hormones have settled and they’ve finished growing, usually around two years of age.” One respondent went further and wrote,

*I have read that it’s best that dogs are desexed after their growth plates have closed, around 18 months which is best for the dog/s if the owner is responsible. However, that’s not always the case, so it would be great to see ovary-sparing spay/vasectomy rather than traditional ops on younger dog/s so they can keep their hormones needed for correct growth.*

The statement regarding the use of behavior–modifying collars elicited a diverse range of responses, with 64.6% either agreeing or strongly agreeing that they should not be used for training, 20.6% selecting neutral, and the remaining respondents supporting their use to some degree. Some of the respondents supported the use of citronella collars, leaving comments such as the following: “I don’t support shock collars or check chains, but used a citronella collar on a former dog of ours as we had had multiple complaints about barking to the council and they already had shelter, food and another dog for company etc., and it was that or rehoming,” and “I would not use shock collars but have used citronella collars years ago for problem barking, and it worked AND the dog was not harmed or traumatized.” Other respondents supported the use of shock collars in specific contexts, offering the following comments; “I think it is ok for a qualified trainer to use shock collars”, “For some dogs, it is crucial that they have behavior modifying collars. In some cases, these could be lifesaving”, and “I don’t believe in shock collars for minor behaviors, but in instances where other animals lives may be at risk, i.e., kiwi aversion training, or stock aversion.” One respondent shared the following:

*One of my dogs stopped being aggressive after a single session with a shock collar which we did not continue as it changed his behavior overnight. It took 11 months listening to bullshit about it damaging a dog before we tried it. Changed his and our lives as now he goes on group walks is off-leash and can do all manner of activities before he was not able to be near motorbikes, bikes, children (he now works as a child therapy dog), trolleys, skateboards, men near me and other dogs.*

One respondent captured the sentiments in many of the responses:

*Each of these things is down to the owner. The problem with shock collars or training collars, docking, branding etc. is not a problem with loving owners who aren’t going to injure or let someone injure their pet. I have dogs with docked tails and ears and it’s not been a problem but some people are cheap and cruel. It is the same for training collars, you have an idiot with a shock collar on their dog day in and day out vs someone who has a high quality, level adjustable collar used only for a short period of training.... it’s not a one answer fits all, it’s down to a moronic owner—it comes down to needing a license to own a dog and massive penalties for cruelty etc.*

Gender, ethnicity, age range, having a child or children, having a rural upbringing, and currently living in a town or city appeared to have influenced specific choice selections. In contrast, household income and qualification level did not. In general, a higher percentage of females and town or city-dwellers tended to strongly agree with various statements. In contrast, Māori, those with children, and those who had a rural upbringing tended to offer a higher percentage of neutral responses. Significant differences are reported in Supplementary Table S2.

### 3.3. Pet Owner’s Attitudes and Knowledge towards Cat Care

Respondents were asked to respond with their level of agreement about statements regarding cat care (Table 2, Supplementary Figure S2). For each statement, there were between 2238 and 2257 responses. Table 2 shows that nearly all respondents (>90%) either agreed or strongly agreed that cats should have access to adequate housing, be desexed, and have up-to-date flea and worm treatments. Most of the respondents (>80%) also agreed or strongly agreed with up-to-date vaccinations, microchipping, and regular veterinarian checks and disagreed or strongly disagreed with cats having their appearance modified.

Of the respondents, 187 provided additional comments to clarify their choice selection. Many comments were left regarding regular veterinarian checks, up-to-date vaccinations, and flea and worm treatments, with all expressing a similar theme that these should only occur when needed. One respondent articulated this attitude by saying, “Each animal needs to be treated as an individual; some have very different needs to others,” while another said, “Flea and worm when required. Vaccinations only required if going into catteries/offsite.” The issue of parasite resistance was often highlighted in the comments. The cost of visiting a veterinarian was mentioned in several comments and was viewed as excessive and prohibitive; for example, “Veterinarians don’t necessarily offer the best solutions for your pet. Too many are for money-making and not what’s best for the animal. Some are just daylight robbers as far as I’m concerned,” and “Veterinarian costs need to be lowered so people can afford to take their whānau there.” “Whānau”, the Māori word for family, expresses that this owner views pets as family members.

A strong theme was also evident in the comments that there should be “Desexing for all pets, except for show/breeding cat/s.” One respondent further stated that the “[l]aw needs to change to ensure all pet cats are desexed unless you are a registered breeder. We have so many unwanted kittens and cats suffering needlessly in New Zealand.” Likewise, there was a strong theme that pet cats should be microchipped. This and the previous theme are reflected in the statement that “all cats should be desexed and microchipped.” One respondent stated that “[c]ats should be microchipped so that if they are hit by a car, the owners can be contacted or so that if the cat is in inappropriate areas hunting wildlife, it can be caught, and the owners notified so they can keep the cat confined.”

Of the respondents, 87% did not feel that pet cats should modify their appearance. Others felt that it was acceptable in certain contexts, for example, “if ‘appearance modification’ is for health reasons”. Another emergent theme was that tattooing was acceptable for reasons of animal and desexed status identification or to help prevent or delay skin cancer. This theme is reflected in the following comments: “Tattooing is sometimes of significant benefit for identifying if a stray cat has been desexed (generally done under GA), but I do not agree with modifications for cosmetic reasons,”; “Tattooing seems more acceptable if done under anesthesia in some situations (e.g., identification). In all cases, tattooing or dying fur for style should be banned”, and “Tattooing on cats’ ears can help prevent/delay skin cancer”. All those respondents who chose to comment about breeding for specific looks expressed a similar opinion to the following: “Be bred for certain looks: agree if they are beneficial to the animal and not detrimental to their health”.

Characteristics of owners, including gender, ethnicity, age range, having a child or children, having a rural upbringing, and currently living in a town or city, appeared to have influenced specific choice selections about the care of pet cats. Unlike dog owners, owners of cats with different household incomes and qualification levels varied in some choices. Again, a higher percentage of females and town/city-dwellers selected strong responses, while those with children had a lower percentage of strong responses. Significant differences are reported in Supplementary Table S3. 

Where the same statement was used in the survey for both cats and dogs, the combined percentage of those respondents that either strongly agreed or agreed with each statement was compared. With respect to the appearance modification phrase, which used opposite wording for cats and dogs, the strongly disagree and disagree percentages for cats were compared to the strongly agree and agree percentages for dogs. When compared to cats, a higher percentage (9.3% difference) of respondents thought that dogs should be microchipped, and a lower percentage agreed with not having their appearance modified (6.5% difference) or being desexed (11.3% difference). All other statements were within ± 5% agreement (Supplementary Figure S3).

## 4. Discussion

The current study aimed to explore the attitudes and opinions of NZ pet owners towards various pet care practices and factors associated with these. Attitudes and opinions towards various cat care practices were associated with gender, ethnicity, age range, having children, rural upbringing, and current place of residence, along with household income and qualification level. However, household income and qualification level did not have a significant impact on attitudes and opinions about dog care practices. In general, females and individuals living in towns or cities tended to give a higher percentage of stronger responses, while Māori, those with children, and those with a rural upbringing tended to offer a higher percentage of neutral responses. The reasons for these differences require further study. Nevertheless, interventions promoting responsible pet ownership should consider these factors when being developed. 

The results revealed that a majority of respondents (>90%) agreed on the importance of providing dogs and/or cats with adequate housing, regular worming, flea treatments, microchipping, and vaccinations. While there is limited research in NZ that focuses on worming and flea treatments, existing studies suggest most NZ pet owners have a positive attitude towards having regular veterinary checks for their pets and are generally committed to providing their pets with a high standard of care [14,19,20]. A recent study conducted in Queensland supports the notion that the frequency of veterinarian visits plays a significant role in encouraging dog owners to make informed health-related decisions for their pets [21]. These findings align with overseas studies that have identified veterinarians as the primary source of information about pet health care and zoonoses [22]. In this study, only 85% of dog owners and 80% of cat owners agreed with the need for regular veterinary visits. Thus, a considerable percentage of respondents were either neutral or did not agree that pet cats and dogs should have regular veterinary visits. For both cat and dog care, respondents being Māori, having children, and having a rural upbringing were associated with a higher percentage of neutral responses regarding the need for regular veterinary visits, whereas respondents’ being town or city dwellers was associated with a higher percentage of “strongly agree” responses. Research into the barriers and enablers associated with regular veterinary visits for pets is needed to develop appropriate interventions that ensure pet cats and dogs are getting the professional healthcare they require. Additionally, it is of paramount importance to understand why certain cohorts of the NZ population do not consider regular veterinary visits important, as lack of access to veterinary healthcare teams may negatively impact both animal and human health, especially in the case of zoonotic diseases.

The present study discovered that around 20% of respondents either agreed or held neutral opinions regarding dogs and cats being bred for certain looks, which might indicate a lack of understanding about some of the animal welfare issues associated with some breeding practices. For example, brachycephalic breeds are associated with various anatomical issues, including stenotic nares, elongated soft palate, and other abnormalities that result in various respiratory health issues [23]. Thus, the findings from this study highlight the need for better awareness campaigns by animal welfare organizations or veterinarians to educate and inform about problematic breeding practices [23].

Improved animal welfare was found to be the only acceptable reason for cats and dogs to have their appearance modified. This finding aligns with the NZ Animal Welfare Act of 1999, which prohibits the docking of canines’ tails except in limited circumstances, with the Ministry for Primary Industries stating that ear pruning and tail docking are not permissible practices in NZ [24]. This result also aligns well with the stance of the NZ Companion Animal Council, who assert pet owners should not alter their animal’s appearance for cosmetic reasons [5], likewise agreeing with a number of overseas laws and regulations that prohibit some of these practices [25,26,27]. In the United Kingdom, for instance, tail docking is prohibited except for certain working canine breeds, and ear pruning is prohibited [25]. In Australia, tail docking is prohibited, with the exception of specific working canine breeds [28], and ear cropping is also prohibited. In the United States, tail termination and ear pruning are legal but regulated in some states [29]. Thus, available information suggests that appearance practices are subject to laws and regulations in some countries and that attitudes towards these practices may vary based on cultural and regional factors.

The current study reported varying opinions with regard to the use of behavior-modifying collars. Citronella collars were favored over shock collars for behavior modification. Opponents of the usage of shock collars contend that the delivery of a shock causes dogs to endure needless pain and suffering [30,31]. It has also been argued that the improper usage of such devices by the average dog owner might generate anxiety in dogs since the unpredictability of shock administration affects stress reactions [31,32]. Anecdotally, there is also the possibility of significant misuse by owners who activate the device out of rage [31,33,34]. Moreover, it is argued that the use of shock collars is seen as a “quick fix” for undesirable behaviors when a more thoughtful approach better aligning with learning theory and dog behavior might allow for a more effective and welfare-compatible resolution of undesirable behavior [31,35]. A great deal of research and many welfare organizations discourage the use of shock collars due to their negative welfare implications [31,36]. Currently, the usage of electric shock devices is prohibited in a number of European nations [31,37] but not in the UK [38]. However, in this study, a number of respondents supported the use of shock collars in specific contexts, such as by a qualified trainer to teach dogs to find but not harm kiwi. Studies have shown that the usage of shock collars corrects “self-rewarding” behaviors by penalizing undesirable behavior in a time–appropriate manner [36,39]. In addition, shock collars are indicated to aid the trainer in teaching dogs alternate behavioral responses, and the usage of these devices has a lower risk of long-term welfare issues than other forms of punishment [35]. Nevertheless, a recent review of the evidence concluded that, overall, shock collars are detrimental to dog welfare [31].

Experts in animal welfare identified deficits in owner knowledge as a major concern [40]. In addition, it has been found that pet owners’ knowledge of responsible pet ownership and zoonosis was limited, indicating a need to improve communication between veterinarians and pet owners [22]. This study would suggest that educational interventions designed to inform pet owners’ opinions and influence their attitudes need to target specific cohorts. Additionally, such interventions should be readily accessible and not depend on the pet owner’s frequenting veterinary clinics, requiring outreach approaches by veterinary health care teams.

### Limitations

One of the strengths of the study was that it was an online survey, which allowed for a greater number of respondents than would a written survey. However, self-selected respondents were disproportionately female, and Māori were underrepresented relative to NZ’s demographics. The preponderance of female respondents was anticipated, as this trend is typical of online surveys. Further research should be conducted using a stratified sampling approach to get fair representation from each of the demographics for researchers to better understand what factors are associated with the attitudes and opinions of NZ pet owners about pet care practices.

## 5. Conclusions

The current study explores factors associated with the attitudes and opinions of adult NZ cat and dog owners about various pet care practices. Almost all of the respondents recognize the importance of providing proper housing, regular worming and flea treatments, microchipping, and vaccinations for dogs and cats. Several demographic factors appeared to influence these attitudes, with variations observed between dog and cat owners. Factors such as gender, ethnicity, age, having children, a rural upbringing, and current residence were associated with various pet care attitudes, highlighting the need for demographically appropriate, tailored interventions to enhance responsible pet ownership in specific cohorts of the NZ population. Of note were the potential knowledge gaps concerning the significance of regular veterinary visits and the negative animal welfare issues associated with breeding for particular looks in dogs and cats. The usage of shock collar devices elicited mixed responses, with some supporting their use in specific contexts while others raised concerns about potential harm and misuse. Improved animal welfare was found to be the only acceptable reason for cats and dogs to have their appearance modified. These insights can guide the creation of educational materials aimed at advancing animal welfare and enhancing the public’s understanding of pet-related matters in NZ.

In summary, the article emphasizes some of the complexities that underlie adult NZ pet owners’ attitudes and opinions towards various pet care practices. The findings underscore the need for additional research and consultation to facilitate the co-design of culturally appropriate and effective educational resources and customized strategies to encourage responsible pet care to ensure animals along with their owners, live good lives.

## Figures and Tables

**Table 1:** Percentage of 2019 New Zealand Pet Survey respondents selecting each level of agreement for the “Dogs should…” statements regarding pet care.

Dogs Should:	Strongly Agree	Agree	Total Agree	Neutral	Disagree	Strongly Disagree	Total Disagree
Have access to adequate housing	90.9%	8.5%	99.4%	0.4%	0.1%	0.1%	0.2%
Have up-to-date worming treatment	66.7%	28.2%	94.9%	3.9%	0.8%	0.4%	1.2%
Be microchipped	77.4%	17.4%	94.7%	4.1%	0.8%	0.4%	1.2%
Have up-to-date vaccinations	69.8%	21.5%	91.3%	7.0%	1.4%	0.3%	1.7%
Have up-to-date flea treatments	63.5%	27.3%	90.8%	7.1%	1.5%	0.6%	2.1%
Have regular vet checks	54.5%	30.7%	85.1%	12.7%	1.9%	0.3%	2.1%
Be desexed	65.0%	18.3%	83.2%	14.7%	1.6%	0.5%	2.1%
Not have their appearance modified (e.g., tail docking, ear cropping, tattooing)	68.4%	12.1%	80.4%	7.6%	3.8%	8.1%	11.9%
Not be trained using behavior-modifying collars	43.6%	21.0%	64.6%	20.6%	8.8%	6.1%	14.8%
Be bred for certain looks	0.8%	2.4%	3.2%	17.6%	27.9%	51.3%	79.2%

**Table 2:** Percentage of 2019 New Zealand Pet Survey respondents selecting each level of agreement for the “Cats should…” statements regarding pet care.

Cats Should:	Strongly Agree	Agree	Total Agree	Neutral	Disagree	Strongly Disagree	Total Disagree
Have access to adequate housing	84.7%	13.2%	98.0%	1.6%	0.2%	0.2%	0.4%
Be desexed	81.3%	13.1%	94.5%	5.1%	0.3%	0.1%	0.4%
Have up-to-date worming treatment	67.4%	26.8%	94.2%	4.6%	0.8%	0.4%	1.3%
Have up-to-date flea treatment	66.4%	26.3%	92.7%	5.8%	1.0%	0.6%	1.6%
Have up-to-date vaccinations	63.3%	23.5%	86.8%	11.0%	1.8%	0.4%	2.2%
Be micro-chipped	66.3%	19.2%	85.4%	11.8%	2.1%	0.6%	2.7%
Have regular vet checks	52.2%	28.7%	80.8%	16.4%	2.3%	0.5%	2.8%
Have their appearance modified (e.g., Branding, tattooing)	3.8%	1.0%	4.8%	8.3%	18.7%	68.2%	86.9%
Be bred for particular looks	0.9%	1.9%	2.8%	20.3%	26.5%	50.4%	76.9%

## Data Availability

The data presented in this study are available on request from the corresponding author.

## Supplementary Materials

The following supporting information can be downloaded at https://www.mdpi.com/article/10.3390/vetsci10100606/s1, Supplementary Figure S1. Percentage of 2019 New Zealand Pet Survey respondents selecting each level of agreement for the “Dogs should…” statements regarding pet care; Supplementary Figure S2. Percentage of 2019 New Zealand Pet Survey respondents selecting each level of agreement for the “Cats should…” statements regarding pet care; Supplementary Figure S3. Dog versus cat owner response: Differences in the percentage of positive responses (strongly agree and agree) for each of the pet care statements; Supplementary Table S1: Demographic questions from the Furry whānau wellbeing: Working with local communities for positive pet welfare outcomes survey; Supplementary Table S2. Factors associated with the choice selections made by the 2019 NZ Pet Survey respondents regarding the care of dogs; Supplementary Table S3. Factors associated with the choice selections made by the 2019 NZ Pet Survey respondents regarding the care of cats.
