# Supplementary material for: Pet Owners’ Attitudes and Opinions towards Cat and Dog Care Practices in Aotearoa New Zealand
Source: Vet Sci. 2023 Oct 4;10(10):606. doi: 10.3390/vetsci10100606 (PMC10611160; doi:10.3390/vetsci10100606)
Supplement: Supplementary file 1 [file vetsci-10-00606-s001.zip › vetsci-2614252-supplementary.pdf]

Three supplementary figures and three supplementary tables.

Supplementary Figure S1. Percentage of 2019 New Zealand Pet Survey respondents selecting each level of agreement for the "Dogs should…" statements regarding pet care.

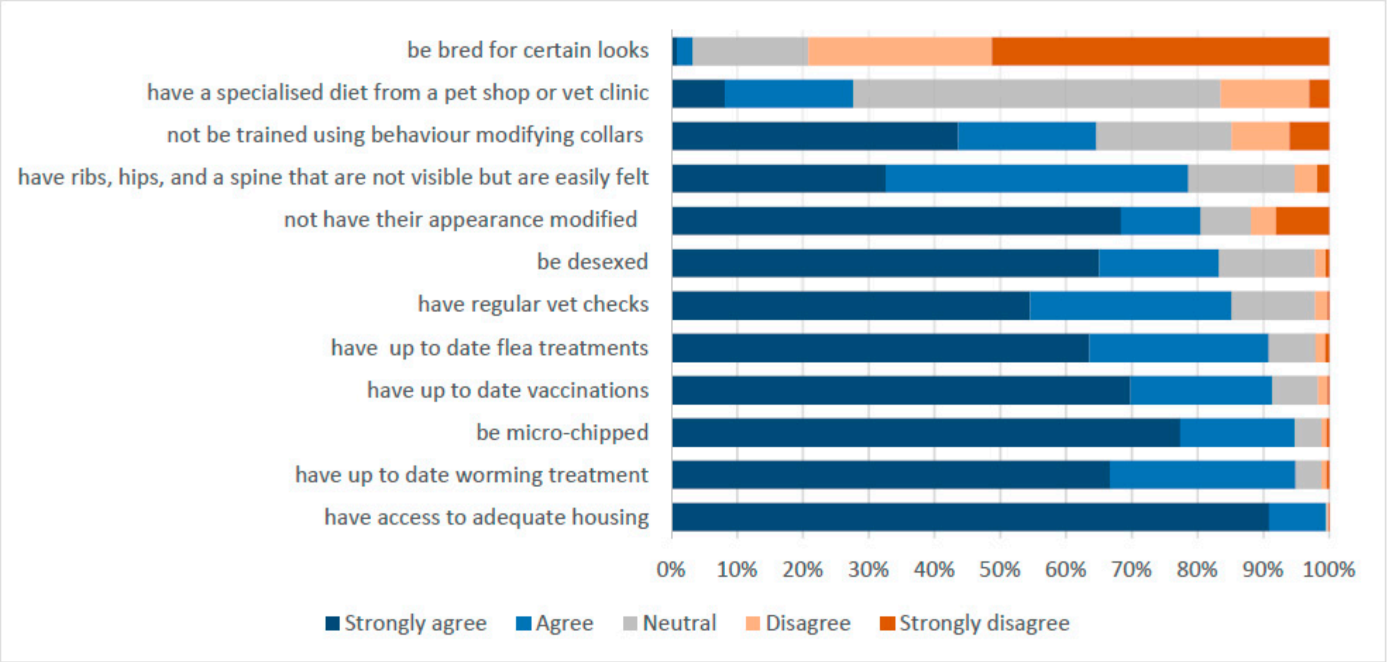

Supplementary Figure S2. Percentage of 2019 New Zealand Pet Survey respondents selecting each level of agreement for the "Cats should…" statements regarding pet care.

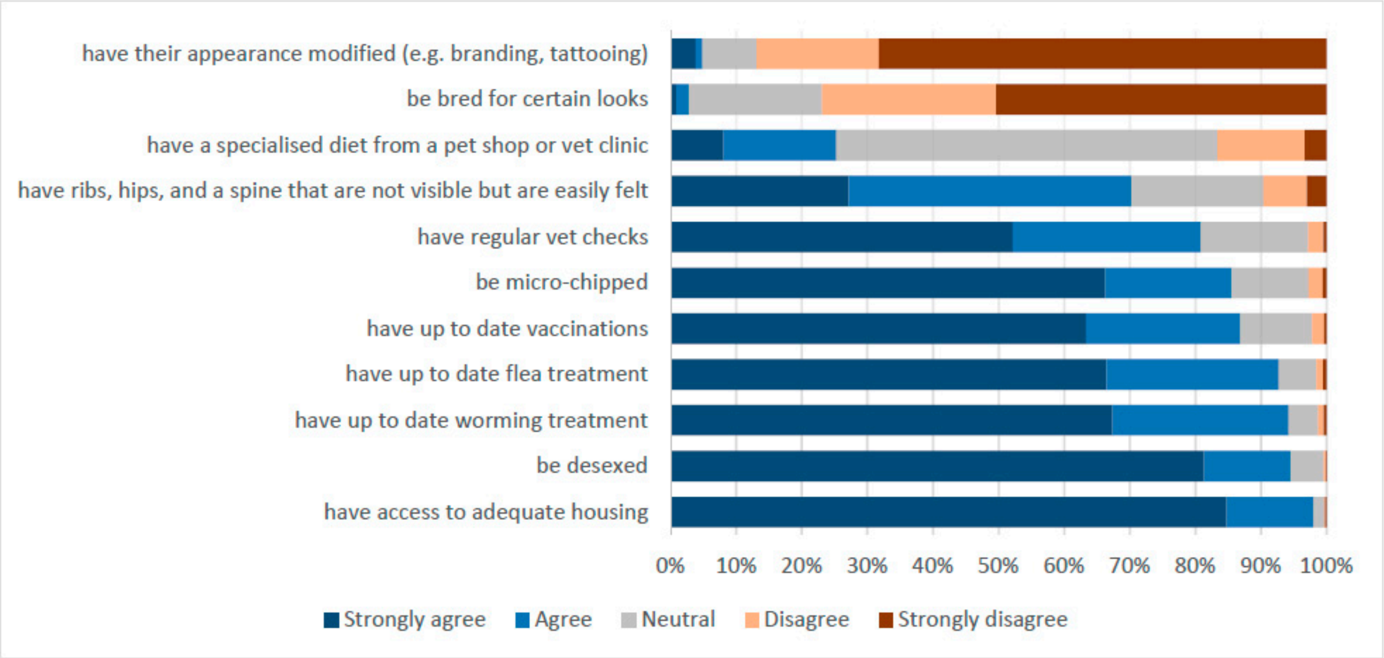

Supplementary Figure S3. Dog versus cat owner response: Differences in the percentage of positive responses (strongly agree and agree[*]*) for each of the pet care statements.*

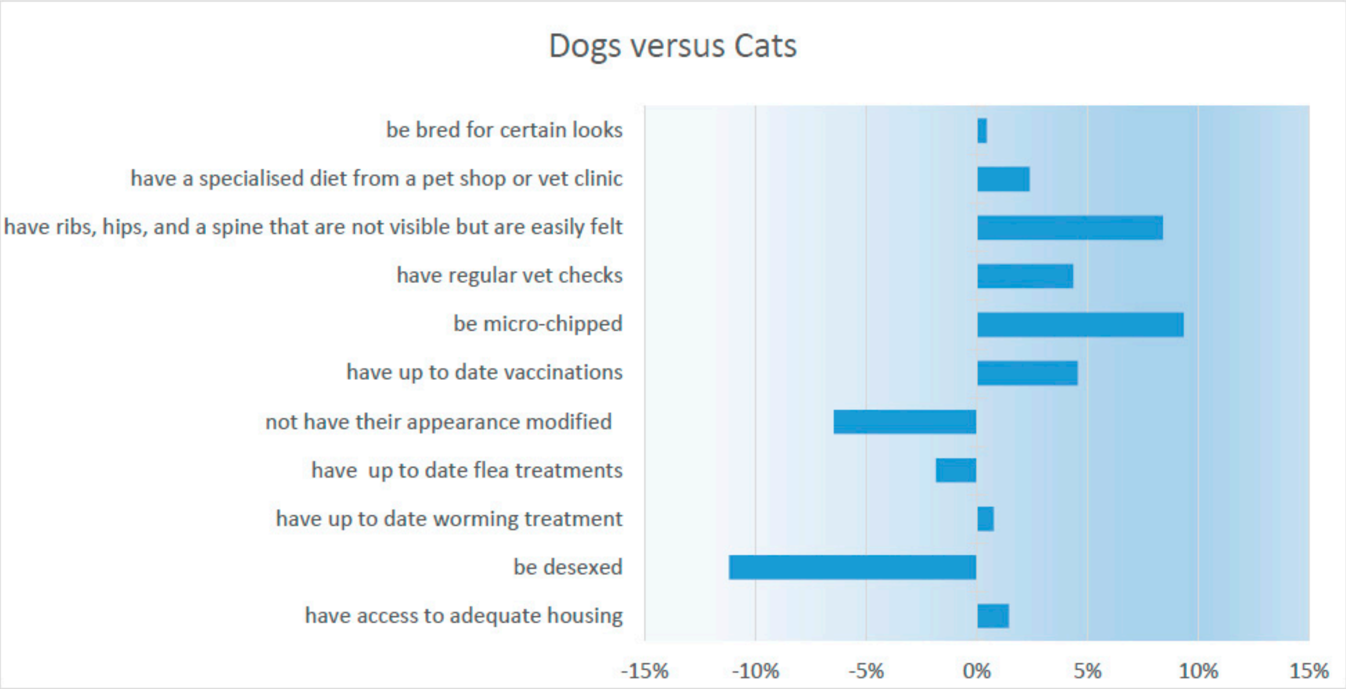

[*] *The appearance modification phrase for cats was "Should have their appearance modified" therefore the strongly disagree and disagree percentages were compared to the strongly agree and agree percentages for dogs where the phrase was "Should not have their appearance modified".*

Supplementary Table S1: Demographic questions from the Furry whānau wellbeing: Working with local communities for positive pet welfare outcomes survey.

Question	Possible Responses
Are you?	Female, Male, Gender Diverse
To which ethnic group do you belong? Tick as many as apply:	NZ European, Other European, Māori, Samoan, Cook Island Māori, Tongan, Niuean, Chinese, Indian, Don't know, Other (please specify)
To which age range do you belong?	18-24 years, 25-34 years, 35-44 years, 45-54 years, 55-64 years, 65-74 years, 75-84 years, 85 years and over
In which region do you currently live?	Northland, Auckland, Bay of Plenty, Waikato, Taranaki, Gisborne, Hawke's Bay, Manawatu-Wanganui, Wellington, Nelson, Marlborough, Tasman, West Coast, Canterbury, Otago, Southland, Other (please specify)
In the last 12 months, what was your total household income?	Less than $14,000, $14,001-48,000, $48,001-70,000, $70,001-100,000, Over $100,000, Would rather not say
What is your highest completed qualification?	National Certificate level 1, National Certificate level 2, National Certificate level 3, National Certificate level 4, Trade certificate, Diploma or Certificate level 5, Advanced trade certificate, Diploma or Certificate level 6, Teachers Certificate or Diploma, Nursing Diploma, Bachelor Degree, Bachelor (Hons), Postgraduate Certificate/Diploma, Master's Degree, PhD, Other (please specify)
Including yourself, how many adults (over 18 years of age) live at your usual address?	0, 1, 2, 3, Other (please specify)
How many children under 18 years of age live at your usual address?	0, 1, 2, 3, 4, 5, Other (please specify)
Were you born in New Zealand?	Yes, No
Where were you brought up? Tick as many as apply:	In a town or city, On a farm or rurally, On a lifestyle block, Other (please specify)
Where do you live now?	In a town or city, On a farm or rurally, On a lifestyle block, Other (please specify)
Do you own a dog?	Yes, No
How many dogs do you own?	1, 2, 3, 4, Other (please specify)
Do you own a cat?	Yes, No
How many cats do you own?	1, 2, 3, 4, Other (please specify)

Supplementary Table S2. Factors associated with the choice selections made by the 2019 NZ Pet Survey respondents regarding the care of dogs.

Dogs should:	Gender (female versus male)	Ethnicity (Māori compared to other ethnicities)	Age range	Children (with versus without)	Rural upbringing	Town/city dwelling
Have regular vet checks		Higher % neutral		Lower % strongly agree; higher % neutral	Lower % strongly agree; higher % neutral	Higher % strongly agree; lower % neutral
Have up to date vaccinations	Higher % strongly agree; lower % agree	Higher % neutral	18-24 and 25-34 age range: Higher % strongly agree; lower % agree	Lower % strongly agree; higher % neutral	lower % disagree	Higher % strongly agree; lower % agree and neutral
Have up to date flea treatments			18-24 age range: Higher % strongly agree; lower % agree	Lower % strongly agree; higher % agree		Higher % strongly agree; lower % agree, neutral and disagree
Have up to date worming treatment			18-24 age range: Higher % strongly agree; lower % agree			Higher % strongly agree; lower % agree and disagree
Be desexed	Higher % strongly agree; lower % agree and neural	Lower % strongly agree; higher % neutral	18-24 and 25-34 age range: Lower % strongly agree; higher % neutral	Higher % neutral and disagree	Lower % strongly agree; higher % neutral	Higher % strongly agree; lower % disagree
Be micro-chipped		Lower % strongly agree; higher % neutral		Lower % strongly agree; higher % agree and neutral		Higher % strongly agree; lower % agree and neutral
Be bred for certain looks				Higher % neutral, lower % strongly disagree		Lower % agree
NOT have their appearance modified	Higher % strongly agree; lower % neural	Lower % strongly agree; higher % neutral				
NOT be trained using behaviour-modifying collars	Higher % strongly agree; lower % neural and disagree		75 - 84 age range: higher % strongly disagree compared to 18-24, 25-34 and 35-44	Lower % strongly agree; higher % neutral	Lower % strongly agree; higher % neutral	Higher % strongly agree; lower % neutral
Have access to adequate housing	Higher % strongly agree; lower % agree and neutral			Lower % strongly agree; higher % agree		

Supplementary Table S3. Factors associated with the choice selections made by the 2019 NZ Pet Survey respondents regarding the care of cats.

Cats should:	Gender (female versus male)	Ethnicity (Māori vs non-Māori)	Age range	Household income	Qualification level	Children (with versus without)	Rural upbringing	Town/city dwelling
Have regular vet checks		Higher % neutral	35-44: Higher % neutral than 18-24- and 25-34-year age ranges			Lower % strongly agree; higher % neutral and disagree	Lower % strongly agree; higher % neutral	Higher % strongly agree, lower % neutral and disagree
Have up to date vaccinations		Higher % neutral	18-24: Higher % strongly agree and lower % neutral than 35-44, 45-54, 55-64			Lower % strongly agree; higher % neutral		Higher % strongly agree, lower % agree and neutral
Have up to date flea treatments			18-24: Higher % strongly agree and lower % agree than 35-44, 45-54, 55-64	48-70K: Higher % strongly agree and lower neutral than 70-100K		Lower % strongly agree; higher % agree and disagree		Higher % strongly agree, lower % agree, neutral and disagree
Have up to date worming treatment	Higher % strongly agree; lower % neutral and disagree		18-24: Higher % strongly agree and lower % agree than 35-44, 45-54, 55-64	48-70K: Higher % strongly agree than 70-100K		Lower % strongly agree; higher % disagree		Higher % strongly agree, lower % agree and disagree
Be desexed	Higher % strongly agree; lower % agree	Lower % strongly agree; higher % neutral	18-24: Lower % strongly agree than 25-34, 35-44, 45-54, 65-74	48-70K: Higher % strongly agree and lower neutral than 70-100K				Higher % strongly agree, lower % agree
Be micro-chipped		Lower % strongly agree; higher % neutral	18-24 and 25-34: Higher % strongly agree than 55-64		>level 7: higher % strongly agree	Lower % strongly agree; higher % agree, neutral and disagree		Higher % strongly agree, lower % agree and neutral
Be bred for certain looks	Higher % strongly disagree, lower % neutral and agree					Lower % strongly disagree; higher % disagree and neutral		
Have their appearance modified						Higher % disagree, lower % strongly agree		
Have access to adequate housing	Higher % strongly agree; lower % neutral and	Lower % strongly agree				Lower % strongly agree; higher %		

	strongly disagree					agree and neutral		
